# Osteotomies around the knee lead to corresponding frontal realignment of the ankle

**DOI:** 10.1007/s00590-021-03016-x

**Published:** 2021-06-04

**Authors:** Christian Konrads, Alexander Eis, Sufian S. Ahmad, Ulrich Stöckle, Stefan Döbele

**Affiliations:** 1 Department for Trauma and Reconstructive Surgery, BG Klinik, University of Tübingen, Tübingen, Germany; 2 Center for Musculoskeletal Surgery, Charité - University Medical Center Berlin, Berlin, Germany

**Keywords:** Deformity, Osteotomies, Realignment, Valgisation, Varisation

## Abstract

**Introduction:**

Despite the fact that osteotomies around the knee represent well-established treatment options for the redistribution of loads and forces within and around the knee joint, unforeseen effects of these osteotomies on the ankle are still to be better understood. It was therefore the aim of this study to determine the influence of osteotomies around the knee on the coronal alignment of the ankle. We hypothesize that osteotomies around the knee for correction of genu varum or valgum lead to a change of the ankle orientation in the frontal plane by valgisation or varisation.

**Materials and methods:**

Long-leg standing radiographs of 154 consecutive patients undergoing valgisation or varisation osteotomy around the knee in 2017 were obtained and utilized for the purpose of this study. Postoperative radiographs were obtained after union at the osteotomy site. The hip knee ankle angle (HKA), the mechanical lateral distal femur angle (mLDFA), the mechanical medial proximal tibia angle (mMPTA) and five angles around the ankle were measured. Comparison between means was performed using the Wilcoxon-Mann–Whitney test.

**Results:**

One hundred fifty-four patients (96 males, 58 females) underwent osteotomies around the knee for coronal realignment. The mean age was 51 ± 11 years. Correction osteotomies consisted of 73 HTO, 54 DFOs, and 27 double level osteotomies. Of all osteotomies, 118 were for valgisation and 36 for varisation. For valgisation osteotomies, the mean HKA changed from 5.8° ± 2.9° preoperatively to − 0.9° ± 2.5° postoperatively, whereas the mMPTA changed from 85.9° ± 2.7° to 90.7° ± 3.1° and the malleolar-horizontal-orientation-angle (MHA) changed from 16.4° ± 4.2° to 10.9° ± 4.2°. For varisation osteotomies, the mean HKA changed from − 4.3° ± 3.7° to 1.1° ± 2.2° postoperatively, whereas the mLDFA changed from 85.7° ± 2.2° to 89.3° ± 2.3° and the MHA changed from 8.8° ± 5.1° to 11.2° ± 3.2°.

**Conclusion:**

Osteotomies around the knee for correction of coronal limb alignment not only lead to lateralization or medialization of the weight-bearing line at the knee but also lead to a coronal reorientation of the ankle. This can be measured at the ankle using the MHA. When planning an osteotomy around the knee for correction of genu varum or valgum, the ankle should also be appreciated—especially in patients with preexisting deformities, ligament instabilities, or joint degeneration around the ankle.

## Introduction

Osteotomies around the knee represent powerful modalities for the treatment of bony deformities and degenerative joint disease [1–4]. The intended effects of these osteotomies act on joints by redistributing loads and force vectors [5, 6]. Despite the fact that osteotomies around the knee represent well-established treatment options for the redistribution of loads and forces within and around the knee joint, unforeseen effects of these osteotomies on the ankle are still to be better understood. Although osteotomies around the knee are successful orthopaedic standard procedures, it is not known to what extent coronal ankle alignment might be intentionally or unintentionally altered.

It was therefore the aim of this study to determine the influence of osteotomies around the knee on the coronal alignment of the ankle. We hypothesize that osteotomies around the knee for correction of genu varum or valgum lead to a change of the ankle orientation in the frontal plane by valgisation or varisation. This new knowledge would help to treat patient better by improving the planning of osteotomies and avoiding unwanted effects on the adjacent ankle joint.

## Patients and methods

**Fig. 1:**
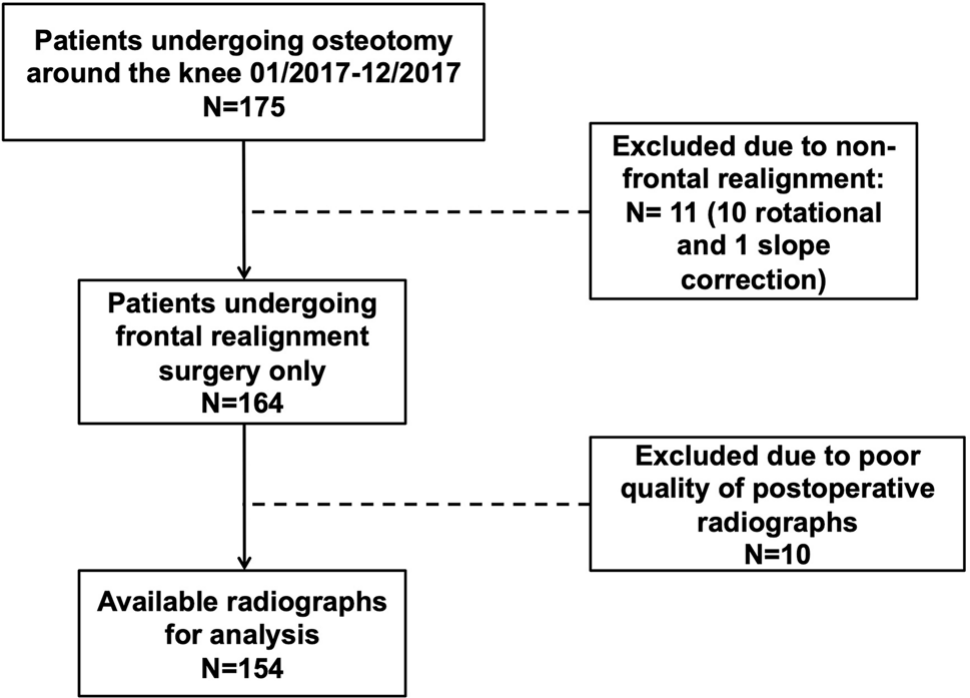
Flowchart demonstrating inclusion

The patient cohort included 154 knees of 154 patients undergoing osteotomies around the knee due to bony malalignment and corresponding symptoms. The mean age was 51 ± 11 years. There were 96 male and 58 female patients. All osteotomies performed were around the knee and included 73 high tibial osteotomies (HTO), 54 distal femur osteotomies (DFO) and 27 double level osteotomies. Of all osteotomies, 118 were valgisation osteotomies and 36 were varisation osteotomies. All consecutive patients were treated in a single center in the year 2017. Patients were excluded, if a multiple plane correction was performed, no magnification device was present on the postoperative radiograph, or image quality was inferior. Considering the above criteria, 154 knees of 154 patients undergoing osteotomy were considered eligible for retrospective data retrieval and inclusion in the study (Fig. 1). Ethical approval was received for the conduction of this study (421/2020BO).

All osteotomies were planned using a landmark based deformity analysis [7, 8]. A high tibial osteotomy was performed as described by Staubli and Lobenhoffer using a TomoFix MHT plate fixator (DePuy Synthes, Solothurn, Switzerland) [9–11]. Distal femoral osteotomy was performed using a medial subvastus approach and the technique described by Lobenhoffer [12–14]. For fixation, a TomoFix MDF plate (DePuy Synthes, Solothurn, Switzerland) was used [15]. Double level osteotomy was performed as described by Schröter et al. [16].

Long-leg weight-bearing radiographs were obtained in accordance with Paley using a 1.3 m cassette (Global Imaging Baltimore, MD) [7]. Long leg antero-posterior standing radiographs were obtained with the patient standing in a bipedal stance in front of the long film cassette. The radiography tube was positioned in a distance of 305 cm. The selected film cassette was of sufficient length to include the hips, knees, and ankles. The magnification with this setup was approximately 5%. A calibration device (250 mm steel ball) was used to calibrate the radiographs. The X-ray beam was centered on the level of the knee joints.

Radiologic technical assistants were instructed to position both legs with the patella centered between the femoral condyles. It was of ultimate importance to ensure a standardized radiography.

**Fig. 2:**
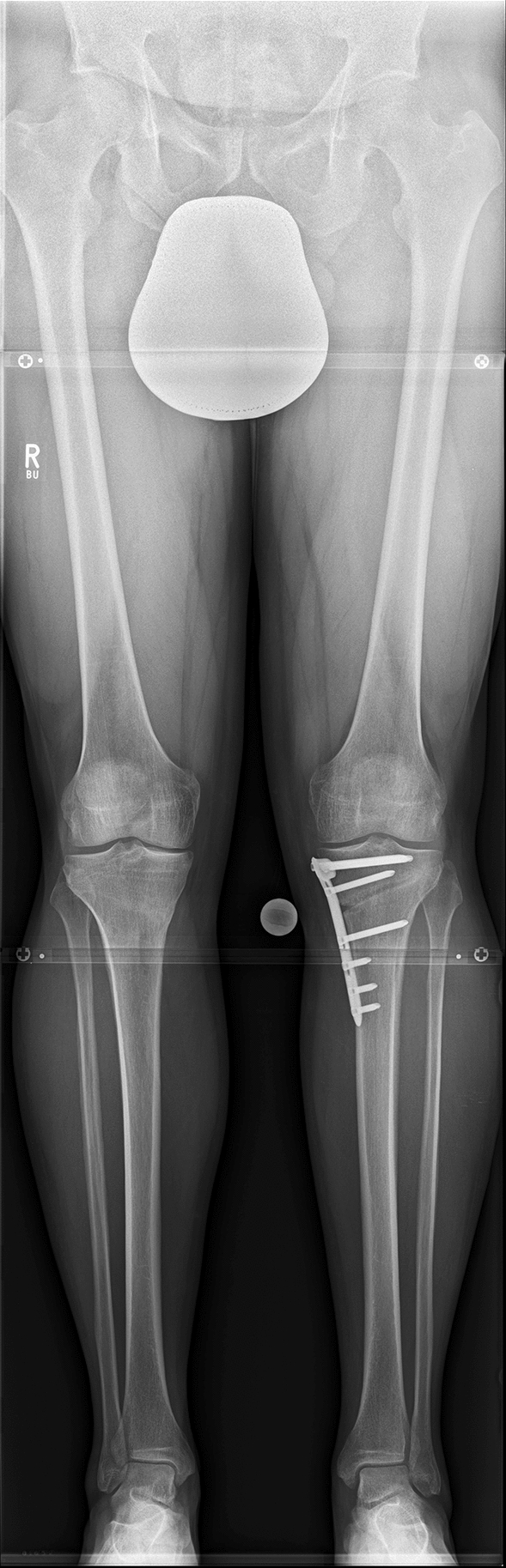
Anteroposterior long-leg weight-bearing radiograph after high tibial osteotomy for valgisation of genu varum

Preoperative radiographs were obtained prior to surgery for planning of the deformity correction and were repeated postoperatively after union at the osteotomy site and recovery of limp-free full weight-bearing (Fig. 2).

**Fig. 3:**
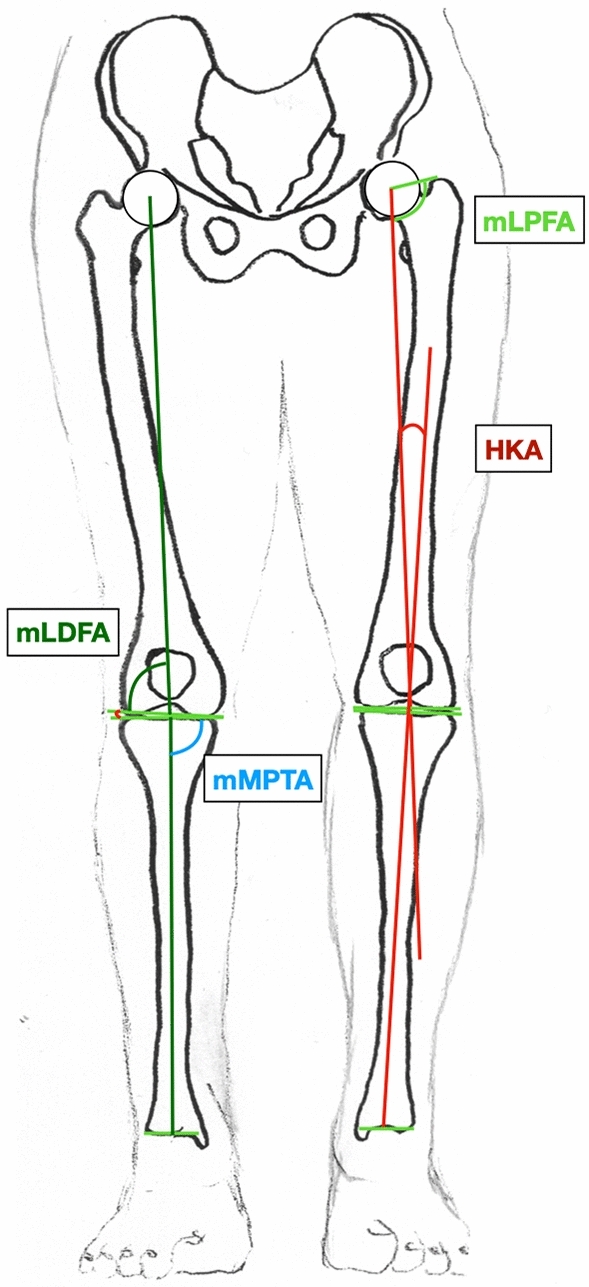
Illustration of the radiographic parameters measured on a long-leg standing X-ray with the knees pointing forward. Measures around the hip and the knee, HKA, Hip Knee Ankle angle, mLDFA, Mechanical lateral distal femoral angle; mLPFA, Mechanical lateral proximal femoral angle, mMPTA, Mechanical medial proximal tibial angle

Radiographic parameters were determined with an accuracy of 0.1 mm using mediCAD® (Hectec, Landshut, Germany). The following parameters were assessed in accordance to Paley [7]:

- Mechanical medial proximal tibial angle (mMPTA)
- Mechanical lateral distal femoral angle (mLDFA)
- Mechanical lateral proximal femoral angle (mLPFA)
- Anatomic Mechanical Angle of the femur (AMA)
- Hip Knee Ankle (HKA) angle, refers to the angle between mechanic axes of the femur and the tibia (Fig. 3). A synonym for HKA is the mechanical tibio-femoral angle (mTFA).

**Fig. 4:**
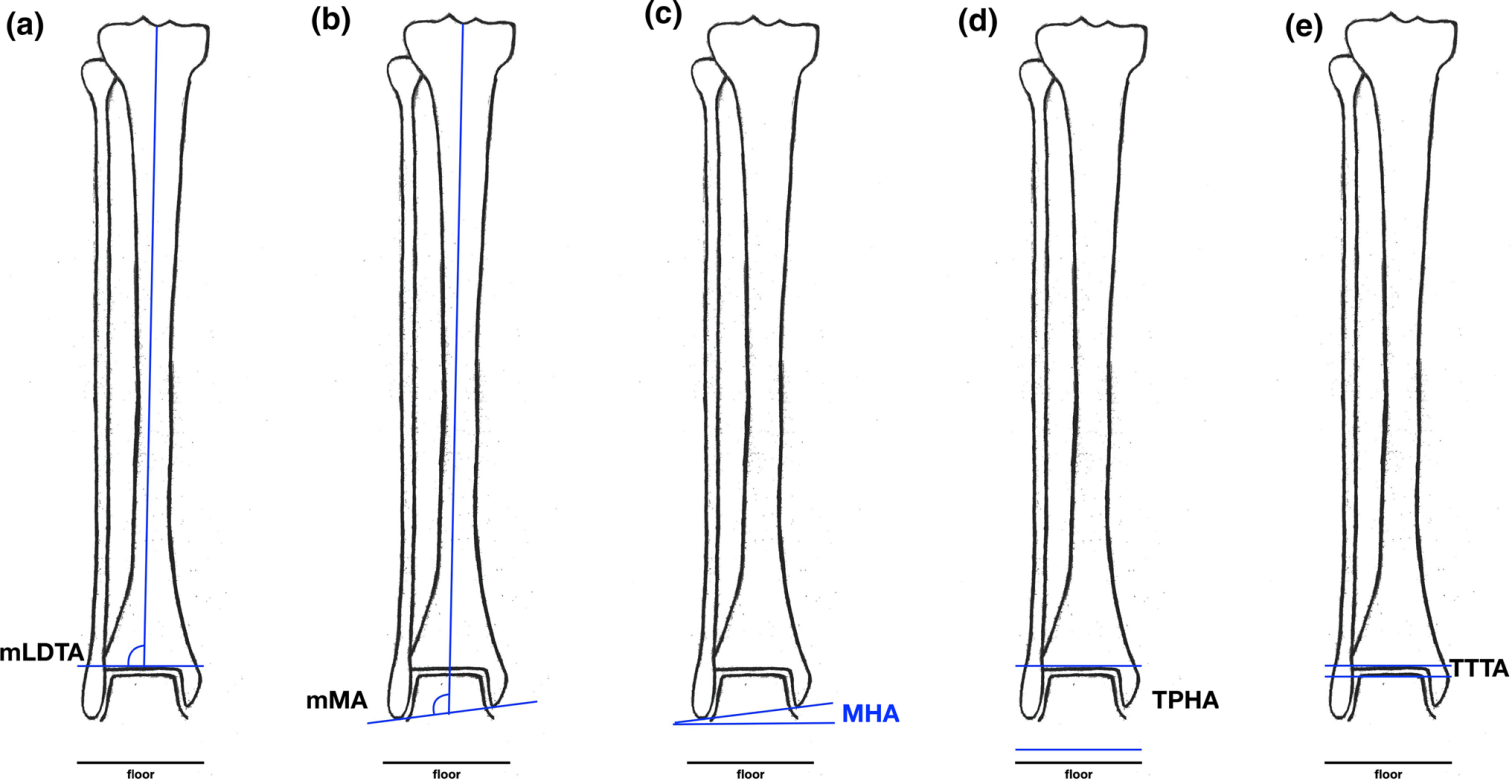
Illustration of the radiographic parameters measured on a long-leg standing X-ray with the knees pointing forward. Measures around the ankle. **a** mLDTA: angle between tibiaplafond and mechanical tibia axis. **b** mMA: angle between malleolar tips and mechanical tibia axis. **c** MHA: angle between malleolar tips and floor. **d** TPHA: angle between tibiaplafond and floor. **e** TTTA: angle between tibio-talar joint surfaces. *mLDTA*, Mechanical Lateral Distal Tibia Angle; *mMA*, Mechanical Malleolar Angle; *MHA*, Malleolar Horizontal Orientation Angle; *TPHA*, Tibia Plafond Horizontal Orientation Angle; *TTTA*, Tibio Talar Tilt Angle

At the level of the ankle, we measured the following radiographic parameters (Fig. 4):

- Mechanical Lateral Distal Tibia Angle (mLDTA)
- Mechanical Malleolar Angle (mMA)
- Malleolar Horizontal Orientation Angle (MHA)
- Tibia Plafond Horizontal Orientation Angle (TPHA)
- Tibio Talar Tilt Angle (TTTA)

For TPHA and TTTA, we defined positive values > 0° to be varus and negative values < 0° to be valgus.

### Statistical analysis

Continuous variables were presented as mean ± standard deviation or range. Comparison between means was performed using the Wilcoxon test. A *p* value of < 0.05 was considered statistically significant. SPSS version 24 (IBM, Armonk, NY, USA) was used. A posthoc analysis was performed to ensure sufficient power to address the primary research question. Given the sample size of 154 patients, an effect size of 1.7 and an alpha error of 0.05, the power of the study was calculated to be 95%.

## Results

Valgisation or varisation osteotomies around the knee led to significant changes regarding the coronal limb alignment—not only around the knee but also at the ankle. This was demonstrated measuring the HKA, mMPTA/mLDFA, and MHA among other measures as shown in Tables 1 and 2.

**Table 1:** Radiographic measures in patients undergoing valgisation osteotomies around the knee

Radiographic measure [°]	Preoperative Mean ± SD (Range)	Postoperative Mean ± SD (Range)	∆	*p*-value
HKA	5.8 ± 2.9 (3.2–17.4)	-0.87 ± 2.5 (-7.0–6.0)	− 6.7	< 0.0001
mMPTA	85.9 ± 2.7 (77.6–92.9)	90.7 ± 3.1 (77.2–97.3)	4.7	< 0.0001
mLDFA	89.2 ± 1.8 (84.9–94.8)	87.4 ± 2.0 (82.5–92.2)	− 1.8	< 0.0001
mLPFA	88.8 ± 9.6 (80.0–101.9)	88.9 ± 4.9 (77.9–100.0)	0.13	0.011
mLDTA	87.2 ± 3.9 (76.5–99.8)	85.8 ± 3.8 (76.5–95.2)	− 1.4	< 0.0001
mMA	101.1 ± 3.6 (90.0–111.0)	100.4 ± 4.0 (86.1–109.2)	− 0.7	0.019
MHA	16.4 ± 4.2 (3.7–27.8)	10.9 ± 4.2 (-0.2–20.0)	− 5.5	< 0.0001
TPHA	4.2 ± 3.4 (0.0–5.0)	1.0 ± 2.9 (0.0–2.4)	− 3.2	< 0.0001
TTTA	0.0 (0.0–0.0)	0.0 (0.0–0.0)	0.0	n. s

*HKA*, Hip Knee Ankle angle; *mMPTA*, Mechanical Medial Proximal Tibial Angle; *mLDFA*, Mechanical Lateral Distal Femoral Angle; *mLPFA*, Mechanical Lateral Proximal Femoral Angle; *mLDTA*, Mechanical Lateral Distal Tibia Angle; *mMA*, Mechanical Malleolar Angle; *MHA*, Malleolar Horizontal Orientation Angle; *TPHA*, Tibia Plafond Horizontal Orientation Angle; *TTTA*, Tibio Talar Tilt Angle

**Table 2:** Radiographic measures in patients undergoing varisation osteotomies around the knee

Radiographic measure [°]	Preoperative Mean ± SD (Range)	Postoperative Mean ± SD (Range)	∆	*p*-value
HKA	− 4.3 ± 3.7 (-11.5–5.5)	1.1 ± 2.2 (− 3.2–6.7)	5.4	< 0.0001
mMPTA	89.7 ± 2.9 (84.7–96.1)	87.8 ± 2.6 (82.6–92.7)	− 1.9	0.001
mLDFA	85.7 ± 2.2 (81.5–90.1)	89.3 ± 2.3 (83.6–94.2)	3.6	< 0.0001
mLPFA	87.7 ± 6.6 (71.8–103.1)	87.9 ± 6.1 (74.1–101.9)	0.18	0.667
mLDTA	85.9 ± 4.5 (75.1–93.2)	85.7 ± 4.9 (71.0–93.0)	− 0.2	0.424
mMA	98.8 ± 6.5 (78.7–111.2)	98.3 ± 6.4 (78.1–104.0)	− 0.5	0.731
MHA	8.8 ± 5.1 (− 2.5–18.5)	11.2 ± 3.2 (4.9–17.0)	2.3	0.002
TPHA	− 2.8 ± 6.2 (− 13.2–17.7)	0.2 ± 2.8 (− 6.2–10.3)	3.0	< 0.001
TTTA	0.0 (0.0–0.0)	0.0 (0.0–0.0)	0	n. s

*HKA*, Hip Knee Ankle angle; *mMPTA*, Mechanical Medial Proximal Tibial Angle; *mLDFA*, Mechanical Lateral Distal Femoral Angle; *mLPFA*, Mechanical Lateral Proximal Femoral Angle; *mLDTA*, Mechanical Lateral Distal Tibia Angle; *mMA*, Mechanical Malleolar Angle; *MHA*, Malleolar Horizontal Orientation Angle; *TPHA*, Tibia Plafond Horizontal Orientation Angle; *TTTA*, Tibio Talar Tilt Angle

**Fig. 5:**
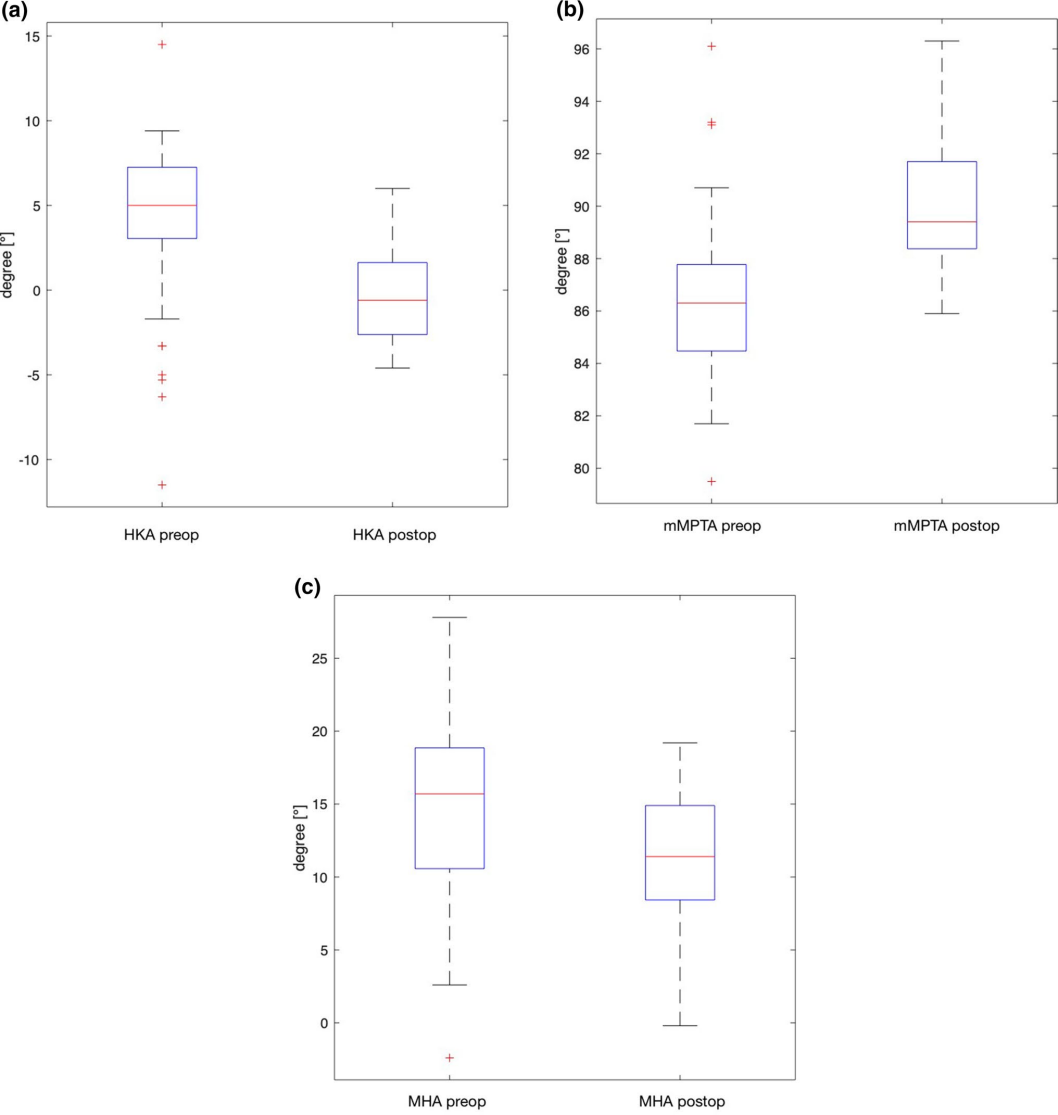
Open wedge high tibial osteotomy for valgisation. **a** Mechanical tibio-femoral angle. **b** Frontal alignment of the proximal tibia. **c** Coronal alignment of the ankle. *HKA*, Hip Knee Ankle angle; *MHA*, Malleolar Horizontal Orientation Angle; *mMPTA*, Mechanical medial proximal tibial angle

**Fig. 6:**
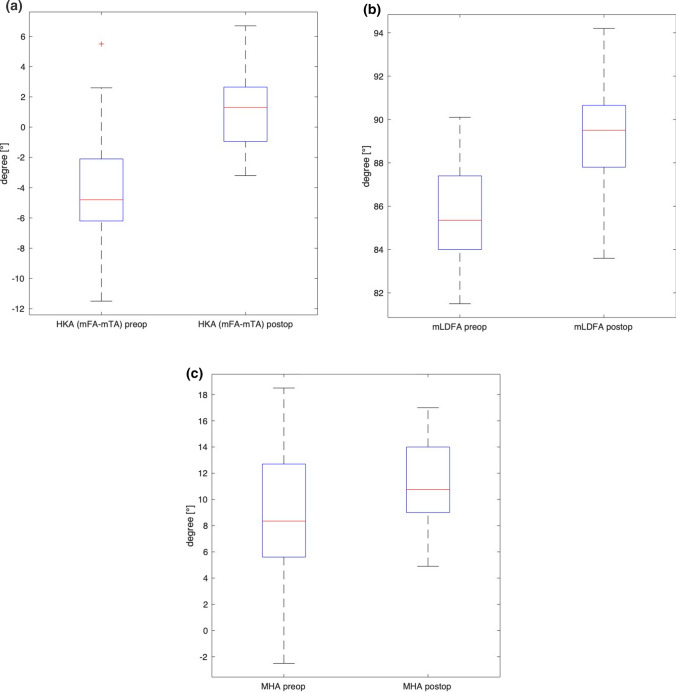
Varisation osteotomies around the knee. **a** Mechanical tibio-femoral angle. **b** Frontal alignment of the distal femur. **c** Coronal alignment of the ankle. *HKA*, Hip Knee Ankle angle; *MHA*, Malleolar Horizontal Orientation Angle; *mLDFA*, Mechanical Lateral Distal Femoral Angle

High tibial open wedge osteotomy for valgisation of the coronal limb alignment led to a corresponding valgisation of the ankle (Fig. 5). Varisation osteotomies around the knee led to corresponding varisation of the ankle (Fig. 6).

## Discussion

We determined the effects of osteotomies around the knee on the corresponding frontal alignment of the ankle. The most important findings of this study demonstrate that an osteotomy around the knee for valgisation or varisation of the long leg axis leads to a reorientation of the ankle in the coronal plane. This can be measured using the MHA (Figs. 4, 5, 6, Tables 1, 2).

The main limitation of the study could be seen in the fact that a 3-dimensional reality has been simplified using 2-dimensional radiography. Addressing this problem would require complex imaging in a functional standing position. Given that the aim of this study was to prove the concept in a feasible standard clinical setting, the authors agreed on the sufficiency of the design chosen in this study.

This work was based on the idea that a change of the weight-bearing line at the knee could alter the coronal orientation of the ankle. Given that a long leg standing X-ray is performed with the knees pointing forward, it can be deduced that the X-ray represents a natural illustration of a standing position of the lower extremity [17–20]. Therefore, one must understand that for an even foot sole contact with the ground after valgisation or varisation osteotomy around the knee, the joints distal to the talus (mainly the subtalar joint) have to adapt by inversion or eversion. This alters not only kinematics, but it must also influence the distribution of joint reaction forces [21].

Regarding the ankle joint, unintentional valgisation or varisation might deteriorate biomechanics especially in patients with ligamentous instability. In the study cohort, the TTTA was 0° in all patients pre- and postoperatively. This could be different and changed by valgisation or varisation in unstable ankle joints, leading to new or aggravated symptoms of instability and pain [19]. In general, unphysiological joint angles should be avoided by meticulous planning and conducting of correction osteotomies [16, 18, 19].

Today, in the era of sub-specialization, communication between the knee surgeon and the foot and ankle surgeon is important. As discussed above, a valgisation or varisation osteotomy around the knee changes the coronal alignment of the ankle, which can be measured using the MHA. The subtalar joint needs to compensate for this with inversion after valgisation osteotomy and eversion after varisation osteotomy in order to secure even foot sole contact with the floor.

This requires a certain mobility in the subtalar joint. Enough range of motion in this region might not be given in patients with an ankle arthrodesis by a nail. In patients with indication for osteotomy around the knee and primary or secondary arthritis of the ankle joints, we would recommend performing the osteotomy around the knee first and then treat the ankle afterwards—especially when indicating ankle arthrodesis [22–27]. With this sequence of surgeries, the surgeon can sustainably accomplish a correct foot position when performing the ankle arthrodesis.

So, not only in symptomatic knees and hips, but also in patients with disease of the ankle, the adjacent joint should be examined and the long-leg axis should be analyzed as part of the routine preoperative workup [28].

## Conclusions

Osteotomies around the knee for correction of coronal limb alignment not only lead to lateralization or medialization of the weight-bearing line at the knee but also lead to a corresponding coronal reorientation of the ankle. This can be measured at the ankle using the MHA and other modalities. When planning an osteotomy around the knee for correction of genu varum or valgum, the ankle should also be appreciated—especially in patients with preexisting deformities, ligament instabilities, or joint degeneration around the ankle.

## Data Availability

The datasets generated and analyzed during the current study are available from the authors on reasonable request.

## Abbreviations

aLDTA: Anatomic Lateral Distal Tibia Angle
AMA: Anatomic Mechanical Angle of the femur
HKA: Hip Knee Ankle angle
JLCA: Joint line conversion angle
mTFA: Mechanical tibio-femoral angle
mLDFA: Mechanical Lateral Distal Femoral Angle
mLPFA: Mechanical Lateral Proximal Femoral Angle
mMPTA: Mechanical Medial Proximal Tibial Angle
aLDTA: Anatomic Lateral Distal Tibia Angle
mLDTA: Mechanical Lateral Distal Tibia Angle
mMA: Mechanical Malleolar Angle
MHA: Malleolar Horizontal Orientation Angle
SD: Standard Deviation
TPHA: Tibia Plafond Horizontal Orientation Angle
TTTA: Tibio Talar Tilt Angle
